# Compressing Chemistry Reveals Functional Groups

**DOI:** 10.1021/acs.jcim.5c02917

**Published:** 2026-03-18

**Authors:** Ruben Sharma, Ross D. King

**Affiliations:** † Department of Chemical Engineering and Biotechnology, 2152 University of Cambridge, Cambridge CB3 0AS, U.K.; ‡ Department of Computer Science and Engineering, Chalmers University of Technology, Gothenburg S-412 96, Sweden

## Abstract

We introduce the first formal large-scale assessment of the utility of traditional chemical functional groups as used in chemical explanations. Our assessment employs a fundamental principle from computational learning theory: a good compression of data should reveal a good explanation. We introduce an unsupervised learning algorithm based on the Minimum Message Length (MML) principle that searches for substructures that compress around three million biologically relevant molecules. We demonstrate that the discovered substructures contain most human-curated functional groups as well as novel larger patterns with more specific functions. We also run our algorithm on 24 specific bioactivity prediction data sets to discover data set-specific functional groups. Fingerprints constructed from data set-specific functional groups are shown to significantly outperform other fingerprint representations, including the MACCS and Morgan fingerprint, when training ridge regression models on bioactivity regression tasks.

## Graphical abstract

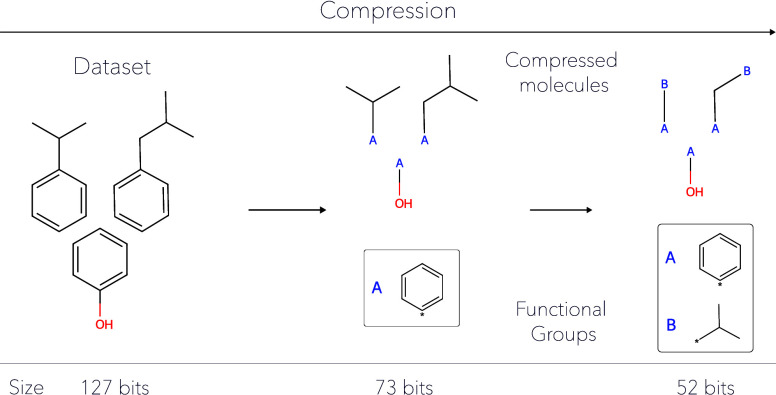

## Introduction

Functional groups are a human-curated set of molecular substructures which are useful for expressing chemical explanations. In organic and biological chemistry molecules are often described as several connected functional groups.^1^ In particular, explanations of chemical and biochemical activity are often described in terms of functional groups. For example, the efficacy of several antibiotic compounds requires the compound to contain the beta-lactam functional group, benzodiazepines are characterized by benzene and diazepine functionalities and NSAIDs contain the propionic acid functional group attached to an aromatic group (see Figure 1).^2–4^ Caching useful substructures that occur in several explanations enables concise descriptions of molecules and their properties. Concise explanations are in line with Occam’s Razor, a preference for simpler (formalized in computer science as shorter) explanations. Specifically, in Solomonoff’s formal theory of inductive inference, the ability of a theory to compress data serves as a formally objective and mathematically optimal measure of the theory’s explanatory and predictive power.^5^ Due to their historical utility, functional groups are often used as a default chemical representation format. In this work we aim to answer the question:

**Figure 1:**
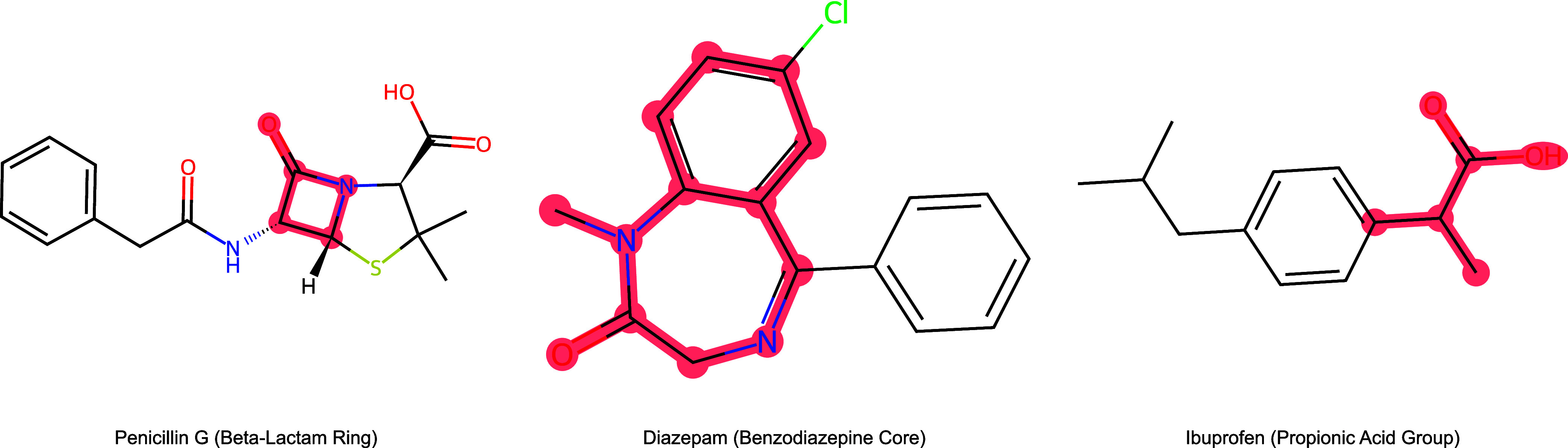
Example drug molecules and necessary functional groups.

*Do substructures that compress a large corpus of biological molecules correspond to human-curated functional groups?*

There exists no canonical functional group set. A positive answer to our research question could establish an objective basis for constructing such a list.

Aside from its intellectual interest, an objective functional group set would enable the development of new chemical similarity measures and representation techniques, such as molecular tokenisation schemes, and more. Further, a method of extracting truly useful substructures from data sets should improve the predictive performance of machine learning and clustering algorithms on chemical data sets.

There are several ways of formulating the compression problem. We choose to employ the Minimum Message Length (MML) principle.^6,7^ MML aims to find the most concise explanation of data, where an explanation is a two-part message consisting of a hypothesis (the first part) followed by the data given that the hypothesis is true (the second part). Under MML, the most concise explanation trades off the complexity of the hypothesis, and how well the hypothesis fits the data. A complex hypothesis (large first part) must fit the data very well (small second part) to be selected over a simple hypothesis (small first part) which fits the data moderately well (moderately sized second part).

In this manuscript, we restrict the types of considered hypotheses to “substructure-based explanations.” Specifically, we model the SMILES representation of a chemical data set as if it were generated by a series of independent draws from a multinomial distribution over SMILES substrings, which correspond to substructures.^8^ We search for the substrings and multinomial probabilities which best compress the data set according to MML. We use MML because the framework automatically chooses the continuous probability parameters of the multinomial distribution. MML’s handling of continuous parameters provides automatic regularisation.

Specifically, we claim:

**Claim 1:** Standard chemical functional groups are objectively useful in general explanations of bioactivity as they emerge during compression of a large corpus of biologically relevant molecules.

**Claim 2:** Substructures that compress a data set are useful features for machine learning tasks.

Overall, our contributions are

**Contribution 1:** We introduce an unsupervised algorithm that identifies a set of compressing substructures from a string data set, in line with the MML principle.

**Contribution 2:** We run our compression algorithm on a data set containing almost three million biologically relevant molecules represented as SMILES strings and show that the discovered substructures validate conventional functional group theory. We provide a list of these substructures.

**Contribution 3:** We use the substructures learned by our compression algorithm to generate chemical fingerprints. We compare the performance of the chemical fingerprints against MACCS fingerprints, Morgan fingerprints, and neural molecular embeddings when learning linear models from bioactivity data.^9^

## Related Work

### Molecular Representation

Most approaches to molecular representation convert chemical structures into vector representations suitable for computation. Vector representations may be continuous, discrete or propositional. We describe each in turn.

A continuous vector representation of a molecule is a sequence of real numbers

1 $$v_{c}\left(m\right)=\left[v_{1},v_{2},...,v_{n}\right]\in R^{n}$$

where the vector ***v***_*c*_(*m*) encodes information derived from the molecule’s structure, composition, or associated data. Examples of continuous vector representations include learned embeddings, where a machine learning model such as a graph neural network learns to map molecular structures into continuous spaces that capture chemical similarity and bioactivity.^10^ In contrast, each vector index in our representation corresponds to a specific feature.

Discrete vector representations are vectors of integer-valued components. A discrete vector representation is defined as

2 $$v_{d}\left(m\right)=\left[d_{1},d_{2},...,d_{n}\right]\in Z^{n}$$

where each *d*_*i*_ usually encodes a count or categorical indicator of a structural or compositional feature. Count-based molecular fingerprints are a common example of discrete vector representations. Each index of a count-based molecular fingerprint corresponds to the integer count of a particular feature, such as a substructure. Binary vector representations are a special case of discrete vector representations, where the components are restricted to elements of the set {0, 1}. Perhaps the most popular discrete vector representation is the Morgan fingerprint.^11^ Morgan fingerprints are constructed by enumerating all substructures within a user-defined radius around each heavy atom, assigning each substructure a unique numerical identifier, and hashing these identifiers into a fixed-length binary vector. The hashing operation, however, does not guarantee a one-to-one mapping between substructures and vector indices. Consequently, some indexes of a discrete vector representation may correspond to multiple substructures. Such events are known as “bit collisions” but are rare in practice. In this report, we learn discrete, count-based vector representations of molecules. In contrast to the Morgan fingerprint, no hashing is applied: Each vector index explicitly represents the count of a unique substructure. Moreover, our approach operates on a substantially smaller set of substructures.

Another popular class of representations is propositional vector representations. Propositional vector representations are vectors with boolean-valued components.

3 $$v_{p}\left(m\right)=\left[p_{1},p_{2},...,p_{n}\right]\in {\left\{True,False\right\}}^{n}$$

Each component of a propositional representation corresponds to a logical proposition, which can either be true or false. For example, propositions may correspond to specific abstractions such as “*the chemical has a 7-membered ring*” or “*the chemical contains an actinide*” or specific groups “*the chemical has the functional group* NC­(O)­N.” One popular propositional representation is the Molecular ACCess Systems keys fingerprint (MACCS) which is of length 166.^9,11^ Each of the 166 entries corresponds to the truth value of a proposition. In contrast our approach uses a discrete vector representation, does not consider propositions which do not correspond to fully specified substructures and has variable length depending on the data set.

### Compression and Pattern Finding

The most relevant prior works include the OSCR and MDLCompress algorithms.^12–14^ Similarly, these algorithms seek to compress the data set using repeating substrings. In contrast to our approach, these algorithms do not calculate the exact length of the compressed data set and rely on heuristics. We also modify our algorithm to extract only those substrings which correspond to valid chemical substructures.

### Identifying Functional Groups

The most similar work to ours is Erten et al.^15^ which also uses an Occamist bias to discover functional groups in an unsupervised fashion. The authors consider a logic programming approach to functional group learning which is more rigorous, but also computationally much more expensive. In contrast to our work, the authors do not run the algorithm on a large scale data set, and do not report all discovered substructures. The authors also do not compare their discovered substructures to existing functional groups, and do not present a method of using them in supervised learning. Another similar work is Ertl.^16^ The authors define functional groups as those which conform to a specified set of rules. The authors use prior chemical knowledge and a programming procedure to automatically partition molecules into functional groups. Conversely, we use no prior chemical knowledge and do not impose any definition regarding the structure of the groups we wish to extract, except those which are syntactically invalid. We simply aim to find groups which may be used to compress a data set in a lossless manner.

## Methodology

We now describe our experimental procedure. To test our claims, we employ two algorithms.

1. FGCompress: This algorithm finds a set of compressing substructures from a data set.
2. FGFingerprinter: This algorithm uses a set of substructures to convert a molecule to a vector representation.

We use the algorithms to test claims 1 and 2. The experiments for each follow

### Experiment 1: Assessing Functional Groups

To assess claim 1, we run FGCompress on a large set of biological molecules (ChEMBL) to extract compressing patterns. Due to computational restrictions we stop searching for patterns after extracting the top 500 most compressive substructures. We then manually assess the extracted substructures to determine if they correspond to human functional groups. We assess structures this way as there is no appropriate canonical human functional group set for comparison. A pictorial representation of the FGCompress algorithm is shown in Figure 2 below. To ensure computational tractability we convert the data set into the SMILES string representation and search for compressing substrings (contiguous lists of SMILES characters) that correspond to chemical substructures. The compression is lossless: together, the compressing substructures and compressed data set can be decoded to reconstruct the original molecules.

**Figure 2:**
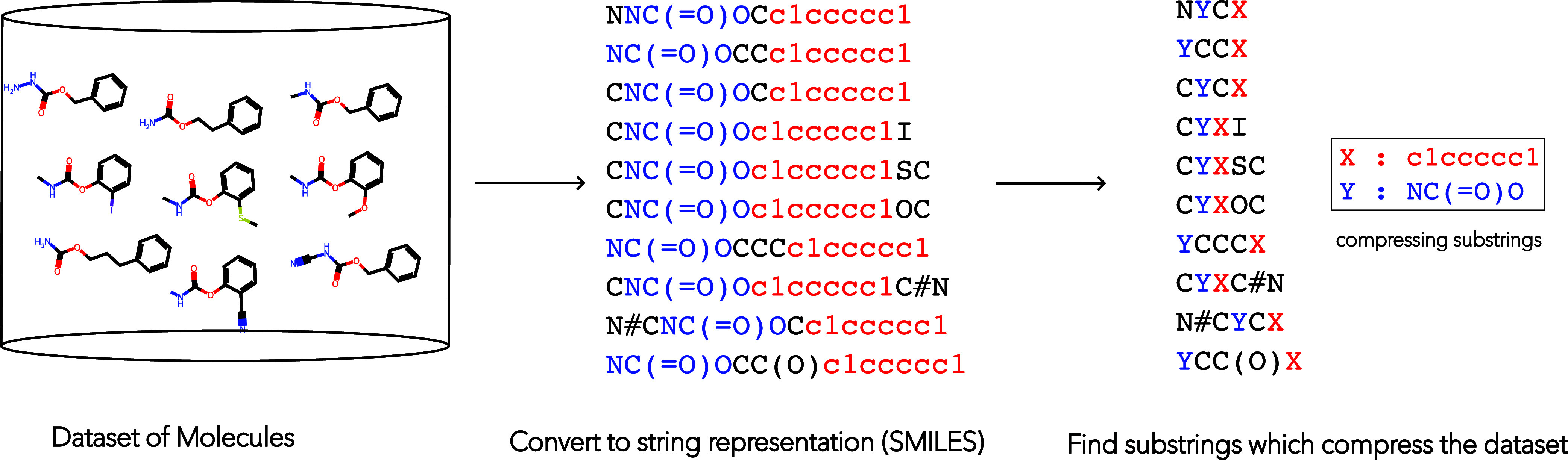
Procedure for extracting substructures (FGCompress).

### Experiment 2: Assessing Learning Benefit of Compressing Groups

To assess claim 2, we independently run FGCompress to completion on the molecules in 24 bioactivity prediction data sets to extract 24 compressing substructure sets. For each data set, the corresponding set of compressing substructures is then used by FGFingerprinter to convert molecules into a count-based fingerprint representation. Representing a molecule as a count based fingerprint is a lossy compression, as it does not contain connectivity information between substructures.

We train ridge regression models to predict IC_50_ values using the FGFingerprinter derived fingerprints. We compare the predictive testing error of the learned regressors against alternative molecular representations: Morgan fingerprints, MACCS fingerprints and neural molecular embeddings from MolFormer-XL.^9,11,17^

A pictorial representation of the experimental procedure is shown in Figure 3. To ensure reproducibility, we repeat each experiment 5 times and take the average accuracy. We conduct statistical tests at *p* < 0.05 to ensure significance, with Benjamini Hochberg corrections.

**Figure 3:**
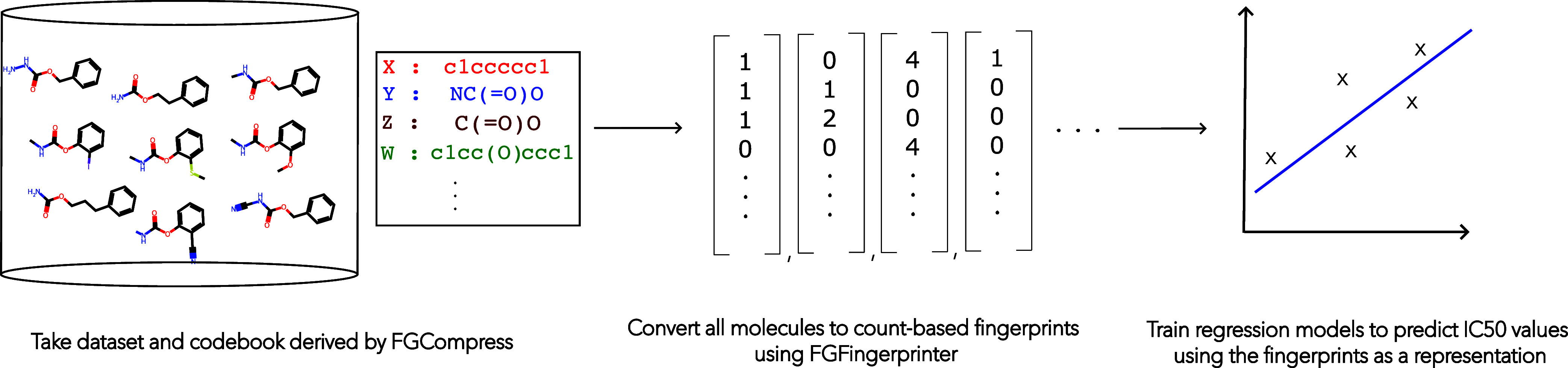
Overall FGFingerprinter Procedure. A data set and codebook are used to convert the molecules in the data set into vectors (count based fingerprints). The vectors are then used to train ridge regression models to predict IC_50_ values.

We describe the introduced algorithms in more depth in the following section.

## Algorithms

To test our claims we require algorithms which (i) identify substructures which compress chemical data sets and (ii) use these compressing substructures in a chemical representation. Our contribution in this section is the introduction of two such algorithms: FGCompress and FGFingerprinter.

### FGCompress

We now detail our substructure discovery algorithm FGCompress. Our algorithm is a greedy search, choosing substructures which best compress the data set at each iteration. The algorithm terminates when no substructure can further compress the data set. The algorithm requires a set of molecules represented as SMILES strings as input. A description of the FGCompress procedure follows.

1. Enumerate all substrings up to a user-specified maximum length in the data set.
2. Filter out substrings that do not correspond to valid chemical substructures. The remaining substrings are termed valid substrings.
3. For each valid substring, compute the total message length obtained if:
  (a) All instances of the substring in the data set are replaced by a single new symbol.
  (b) The new symbol–substring pair is added to the codebook.
  (c) The updated codebook and data set are transmitted as a message.
4. Select the substring that yields the shortest total message length (maximally compresses the data set).
5. If the selected substring reduces the total message length, add it to the codebook and return to step 1.
6. Otherwise, if no new substring can further reduce the message length, terminate and return the current codebook.

If a newly added substring (step 4) contains an existing codebook entry, the count of that entry is reduced accordingly. If the count of an entry reaches zero, the substring is removed from the codebook. A diagrammatic representation of a hypothetical FGCompress run is shown in Figure 4 below.

**Figure 4:**
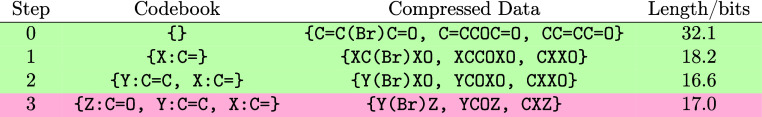
An illustration of an example run of the FGCompress algorithm. At iteration 2, adding the best substring C=O does not further compress the message. The algorithm is then terminated and the codebook at step 2 is taken as final. Length values are invented for illustrative purposes.

At each step, the message length is calculated as the number of bits required to transmit the codebook and data set, modeling molecules as draws from a multinomial distribution specified by the codebook. We note that our algorithm considers substrings which may themselves contain previously compressed substrings. For example, in step 3 of Figure 4 the algorithm assigns the candidate codeword *Z* to the substring XO, which is equivalent to C=O as X:C =. Consequently, even if the algorithm only enumerates substrings up to a small user-specified maximum length, larger substrings can still be extracted by the end of the algorithm. We discuss the effect of the user-specified maximum substring length (step 1 of the FGCompress procedure) with our experimental results.

We describe specific calculation of the message length in the next section.

### FGFingerprinter

This algorithm takes as input the set of compressing substructures from FGCompress and generates a molecular fingerprint. The fingerprint is a fixed length integer vector of counts. Each index of the vector corresponds to a substructure which was discovered during the FGCompress search. To generate a molecular fingerprint for a molecule, FGFingerprinter counts the number of times each substructure occurs in the molecule and sets the corresponding vector index to this count value. We refer to this fingerprint as the MML87 fingerprint, as the FGCompress search procedure relies on the MML87 approximation to calculating the MML codelength.^18^ The MML87 fingerprint is a lossy chemical representation as it does not retain information pertaining to connections between the substructures. A pictorial representation of using a codebook to construct a fingerprint is shown in Figure 5. The larger the set of compressing substructures, the larger the molecular fingerprint vector.

**Figure 5:**
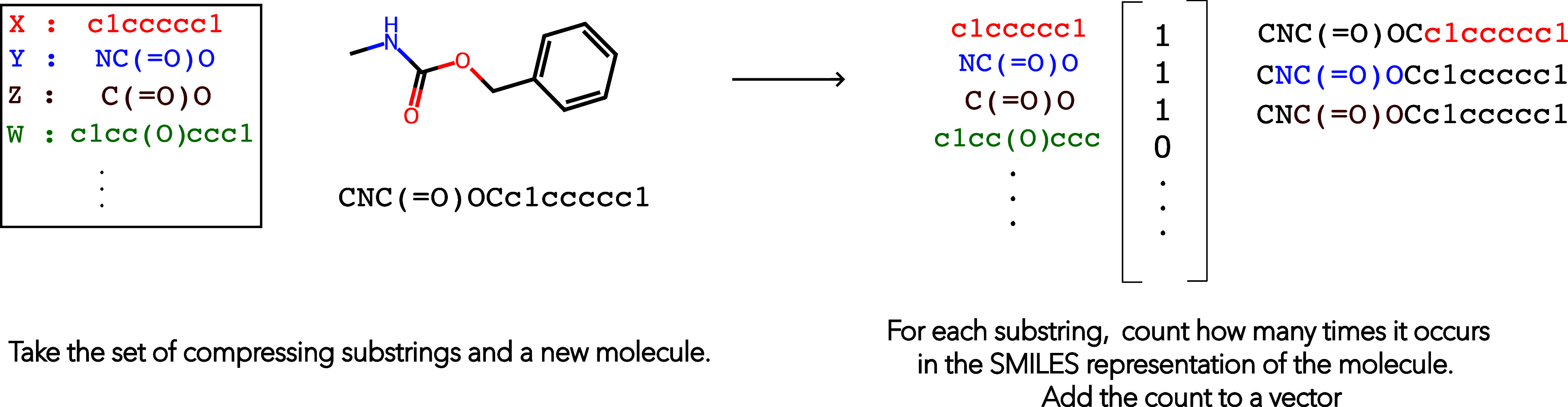
Conversion of a molecule to count-based fingerprint given a codebook.

## Definitions

Definition 1 (**SMILES symbol**). A SMILES symbol is an element of the SMILES alphabet as defined in Weininger.^8^ We denote the SMILES alphabet as Σ.

Definition 2 (**Valid Substring**). Several concatenations of SMILES symbols do not correspond to valid substructures. To ensure our codebook contains as few invalid substructures as possible, we filter the set of all substrings.

The set of all substrings Σ* is the set of all finite strings which can be constructed from Σ. The set of valid substrings *S* = *F*(Σ*) ⊂ Σ* is the set of all strings that satisfy the filter function *F*: Σ* → Σ*. A valid substring is an element of the set of valid substrings *s* ∈ *S*. In this report, *F* filters Σ* for those substrings using the following rules.

- If a substring contains a bracket, then it must also contain the corresponding matching bracket
- If the substring contains a bond character (=,#,-,/,\) or stereogenic center character (@), then it must contain at least one atom connected to this character.
- If the substring contains a number, then it must also contain a second instance of that number (c1cccc not permitted).
- The character is not permitted in any substring.

Informally, a SMILES substring is any contiguous list of SMILES characters that is present in a molecule. For example, given the molecule C­(=O) there are 15 possible substrings.
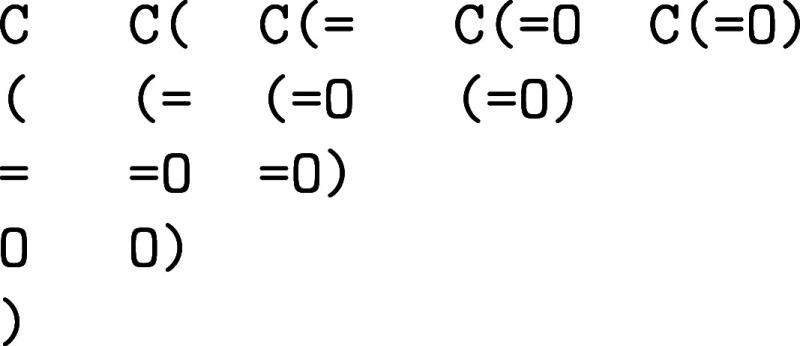


Of which only 8 are valid substrings.
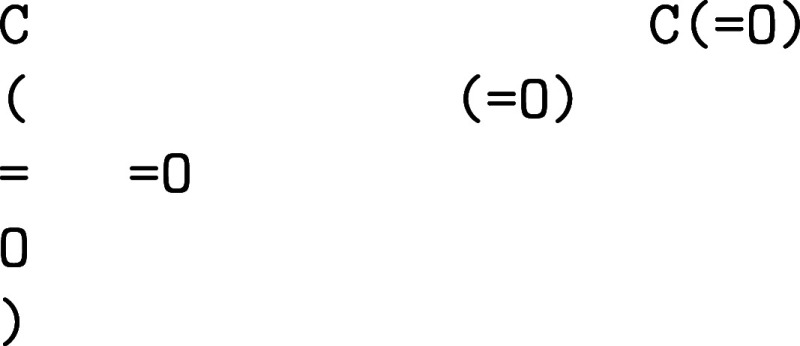


Definition 3 (**Codebook**). A codebook is a set of pairs {(*s*_1_,*p*_1_),...(*s*_*n*_,*p*_*n*_), where *s*_*i*_ ∈ *S* is a valid substring and *p*_*i*_ ∈ [0, 1] is a probability value. The probabilities are constrained such that $\sum_{i=1}^{n}p_{i}=1$ . The codebook is interpreted as a multinomial probability distribution: substring *s*_*i*_ has probability *p*_*i*_ of being drawn randomly, with replacement.

Definition 4 (**Optimal Coding Scheme**). We represent the codebook and compressed data set as binary strings. To ensure our encoding is as concise as possible, we use an optimal coding scheme to represent each part of the message.^19,20^ An optimal coding scheme is designed to minimize the average length of an encoded message. The scheme assigns shorter binary strings to more probable events and long strings to less probable ones. Formally, an event *x* with probability *P*(*x*) is represented as a binary string of length −log_2_ *P*(*x*). Hereon, all log terms are assumed to be base 2. An optimal coding scheme ensures that the average length of the encoded data approaches the entropy of the true symbol generating distribution, the theoretical limit of compression. An optimal code is a code from an optimal coding scheme.

## The Message

We now describe the type of message that we send. The length of the message is used as our measure of compression and guides the search toward maximally compressing substrings. We use the terms codebook, valid substring and optimal coding scheme as defined in the previous section. Before the message is communicated, we assume that the sender and receiver have agreed that the probability of a SMILES symbol occurring in a codebook substring is equal to its relative frequency in the data set. Consequently, the receiver is assumed to already know the symbol frequencies in the data set. This assumption is equivalent to assuming that the receiver is aware of some preliminary analysis on the data set but does not know of a good codebook that compresses the data further. The message therefore consists of this codebook, and the data set compressed by the codebook. An example illustration of the message contents is shown in Figure 6.

**Figure 6:**
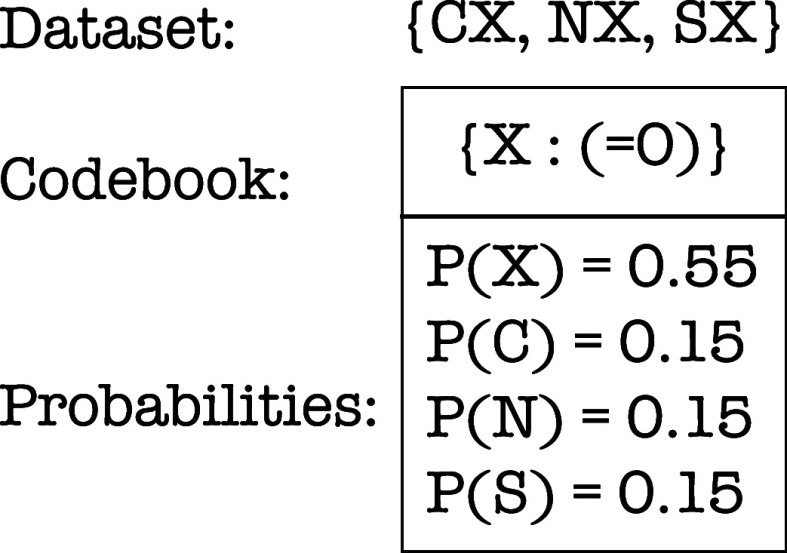
Example message contents for the original data set {C­(=O),N­(=O),S­(=O)}.

### Message Length Calculation

We now describe how we calculate the message length, the quantity we seek to minimize during our search procedure. Conventionally, a message is described in two parts: sending the codebook (and probabilities), and sending the data set. Instead, for clarity we describe the message in three parts. Part 1 communicates the substrings in the codebook. Part 2 sends both the probability values in the codebook and the substring and symbol counts in the data set given part 1. Part 3 communicates the specific sequence of symbols and substrings given parts 1 and 2. We split the message into these three parts because there exists a known expression for part 2 that we use in our implementation. We use the term “vocabulary” to refer to the minimal set of substrings and symbols required to cover the full data set. In Figure 6, the vocabulary is {X,C,N,S}.

The length of the message communicating part 1 is determined by sending each part piecewise. As discussed, the length of communicating part 2 has an already known closed form. The length of communicating part 3 is calculated from the multinomial coefficient: the number of sequences one can make from a data set of length *N*, composed of *i* symbols with frequencies *M*_1_, *M*_2_, ...*M*_*i*_, where all sequences are equally likely. We now describe calculation of the three parts in order.

#### Part 1: Communicating the Substrings

First the sender sends each substring in the codebook. The message that specifies all substrings is structured hierarchically. The sender first specifies the total number of substrings in the codebook, |*H*_*s*_| and then sends the data for each substring sequentially. Let *T* and *s_i_* denote the codewords representing the total number of substrings in the codebook, and the i^th^ substring, respectively. Then the message format is:

*[T]­[s_1_]­[s_2_]...*

Each individual substring is also a two-part message, specifying its length followed by the constituent symbols. Let *l­(s_i_)* and sym*_j_(s*_i_ *)* denote the codewords representing the length of substring i and the jth symbol of substring i. Then the individual substring message is:

*[s_i_]*= *[l­(s_i_)]­[*sym_1_ *(s_i_)]­[*sym_2_ *(s_i_)]*...

All integer values (total substring count and individual substring lengths) are communicated using an integer code. The substring symbols are encoded using their probabilities.

1. **Encoding integer values:** All integers *N* are encoded using the log-star universal code.^21^ This is a special code which allows for specifying any integer in a concise manner. Under the log-star code, the transmission cost for the integer is log  *­(*N*) ≈ log­(*N*) + log log­(*N*) + ... bits.
2. **Encoding substring symbols:** Each symbol *s* in substring *S*_*i*_ is encoded according to their preagreed probabilities of occurrence, as discussed at the beginning of this section. Under an optimal coding scheme, the cost of transmitting symbol *s* is −log_2_ *P*(*s*) bits.

The cost of specifying a single substring *C*_string_(*S*_*i*_) is the sum of the cost of its length and costs of all constituent symbols (eq 4).

4 $$C_{string}\left(S_{i}\right)=\log^{*}\left|S_{i}\right|-\sum_{s\in \Sigma }\left[count\left(s,S_{i}\right)\times \log P\left(s\right)\right]$$

where count­(*s*, *S*_*i*_) is the number of times symbol *s* appears in substring *S*_*i*_.

The total cost of all of the substrings is the cost of specifying the number of total substrings, plus the sum of the costs of each substring (eq 5).

5 $$P_{1}=\log^{*}\left|H_{s}\right|+\sum_{i=1}^{\left|H_{s}\right|}C_{string}\left(S_{i}\right)$$

For example, consider sending the example substring *S*_ex_ = CCCN where *P*(*C*) = 0.5 and *P*(*N*) = 0.25.

- The cost to transmit the length (|*S*_ex_| = 4) is log  *(4) ≈ 2 bits.
- The cost of transmitting the three C symbols is 3 × (−log  0.5) = 3 bits.
- The cost of transmitting the single N symbol is 1 × (−log  0.25) = 2 bits.

The total length *C*(*S*_ex_) is then 2 + 3 + 2 = 7 bits.

#### Part 2: Sending Vocabulary Probabilities and Counts

The part 2 message communicates two pieces of information (i) the underlying probabilities assigned to each item in the vocabulary and (ii) the specific counts of those items observed in the data set. A known approximation to the optimal length of this combined message is given by the MML87 formula for the multinomial distribution. We state this here and refer the reader to Wallace^6^ and Wallace and Freeman^18^ for a full derivation. The length of this part of the message, denoted *P*_2_ is

6 $$P_{2}=\log_{2}\frac{\Gamma \left(N+M\right)}{\Gamma \left(M\right)}+\sum_{m}\log_{2}\frac{1}{\Gamma \left(s_{m}+1\right)}+\frac{1}{2}\log\left(\left(M-1\right)\pi \right)-0.4$$

where *N* is the total number of substrings and symbols in the data set, *M* is the number of distinct substring and symbols, Γ(.) is the gamma function, *s*_*m*_ is the count of the *m* ^*th*^ symbol and π is the standard mathematical constant.

#### Part 3: Specifying the Sequence of the Symbols

While the part 2 submessage specifies the number of symbols and their probabilities, it does not communicate their ordering. The final part of the message encodes the specific sequence of the *N* total substrings and symbols in the encoded data set. The number of unique sequences that can be formed is given by the multinomial coefficient. Assuming all valid orderings are equally likely, then the probability of a single sequence is simply $\frac{1}{number\;of\;unique\;sequences}$ . Under an optimal code, the cost of specifying the specific sequence the negative logarithm of the probability of a specific sequence, denoted *P*_3_. We assume that the receiver knows the number of individual symbols in each SMILES string, so that they can split the received string into the original molecules.

7 $$P_{3}=\log_{2}\frac{N!}{s_{1}!s_{2}!\cdot \cdot \cdot s_{M}!}$$

### Full Message Length

The full message length is the sum of the lengths of the three parts

8 $$M=P_{1}+P_{2}+P_{3}$$

We use eq 8 as our cost function, and implement a search procedure to minimize this value.

## Experimental Questions

We conducted experiments to verify our claims. We first try to answer the question:

**Q1** Do functional groups emerge when compressing a large corpus of biologically relevant molecules? We answer **Q1** by running the FGCompress search procedure on all molecules in the ChEMBL data set for 500 iterations, and caching the discovered substructures. We terminate the algorithm after 500 iterations due to computational constraints. While early termination may prevent the discovery of additional patterns, FGCompress extracts substructures in order of importance, so the top 500 substructures are expected to strongly represent the data set. Unfortunately, there is no canonical list of functional groups to which we could compute a similarity score. Instead, we manually assess the discovered substructures, and note exact and partial matches to human, named functional groups.

To test our claim that the discovered substructures aid learning, we try to answer the question:

**Q2** Are the substructures extracted from compressing the data set useful for learning? We answer **Q2** by generating a unique MML87 fingerprint for each of 24 bioactivity prediction data sets using the FGFingerprinter algorithm. We compare the predictive performance of four fingerprint representations: our MML87 fingerprint, the 166 bit MACCS fingerprint, a Morgan fingerprint (radius 2) hashed to the same length as the corresponding MML87 fingerprint for that specific data set and a continuous neural representation (MoLFormer-XL).^9,11,17^ We train a cross-validated ridge regression model for each data set.^22,23^ We compare the mean squared error of the models on a test set using each fingerprint.

### Data and Experimental Choices

We use canonical SMILES representations as input for all FGCompress runs. We use the following data sets for each experiment.

#### Experiment 1 (Q1)

**Data** We ran the FGCompress algorithm on the ChEMBL data set (release 36), which contains 2,878,135 bioactive molecules.^24^ We chose the ChEMBL data set as the data is high quality and manually curated. **FGCompress** **Parameters** We used a maximum substring enumeration length of 8 and a uniform prior distribution over all substring probabilities via a generalized beta function.

#### Experiment 2 (Q2)

**Data** We ran the FGCompress algorithm on the 24 bioactivity prediction data sets, reported in Cortés-Ciriano and Bender.^25^ These data sets contain IC_50_ data for 24 diverse protein targets and receptors from the ChEMBL database. In their study, the authors state that they only retained IC_50_ values for small molecules for which.

- The activity unit was equal to “nM”
- The activity relationship was equal to “ = ”
- The target type was equal to “SINGLE PROTEIN”
- The organism was equal to *Homo sapiens*.

#### Models

We choose ridge regression models over more expressive models such as random forests, gradient-boosted trees, or neural networks because our goal is to directly evaluate the quality of the substructures. Random forests and gradient-boosted trees implement learned feature transformations on top of the input features, while neural networks construct internal representations in their hidden layers.^26–28^ In contrast, ridge regression provides a purely linear mapping from features to predictions, making it ideally suited for assessing raw representation quality.

#### Hardware and Implementation

We ran all FGCompress experiments on a server with 36 CPU cores and 125 GB RAM. We ran the FGFingerprinter experiments on an M2MacBook Air (8 CPU cores, 16 GB RAM). All experiments were repeated 5 times, using random 75/25 train/test splits, and we report the mean and standard error in each case. Statistical significance was assessed using the Wilcoxon signed-rank test with Benjamini-Hochberg multiple testing corrections at α = 0.05.^29,30^ Significance tests were conducted between each fingerprint representation over all data sets to satisfy the independence assumptions of the tests. Ridge regression models were implemented using scikit-learn.^31^ The ridge α hyperparameter was optimized via leave-one-out cross-validation on the training set, selecting from {0.001, 0.01, 0.1, 1}.

## Results and Discussion

We now present the empirical results to address our experimental questions **Q1** and **Q2**.

### Q1: Functional Group Verification

The full list of substructures discovered by FGCompress may be found in the appendix. In all figures, substructures are listed in order of the iteration at which they were derived. This reflects the explanatory power of the substructure with respect to the data set. The resulting substructures compress the data set by 30% relative to the data set encoded in terms of SMILES symbols only.

Of the 500 substructures, 494 were successfully converted to SMARTS patterns using RDKit. A substructure was considered convertible if it is already a valid SMARTS pattern or becomes valid by adding the wildcard “*” at the beginning, end, or both. Consequently, we conclude that use of the SMILES representation in our procedure did not significantly affect the interpretability of the extracted substrings.

After filtering for substrings that can be converted to valid SMARTS patterns, we further remove substructures that are identical up to numerical relabeling. For example, the substring c2ccccc2 would be removed if c1ccccc1 is already present. After filtering, FGCompress returned 304 substructures. We next analyze the filtered list.

The top 15 substructures found by the algorithm are shown in Figure 7 below. All of these substructures are well-known functional groups. For example, the top five groups include the carbonyl, trifluoromethyl, methyl, amide, benzene functional groups. As ChEMBL is a bioactivity data set we expect functional groups that are strongly represented in the biological literature, such as those which are derivatives of amino acids (substructures 4, 6, 9, 11, 12, 14). Substructures 4 and 9 are identical as they are two SMILES representations of the amide group C­(=O)­N and NC­(=O) that were not caught by our filtering procedure. The duplication of the amide group is a direct artifact of the SMILES representation.

**Figure 7:**
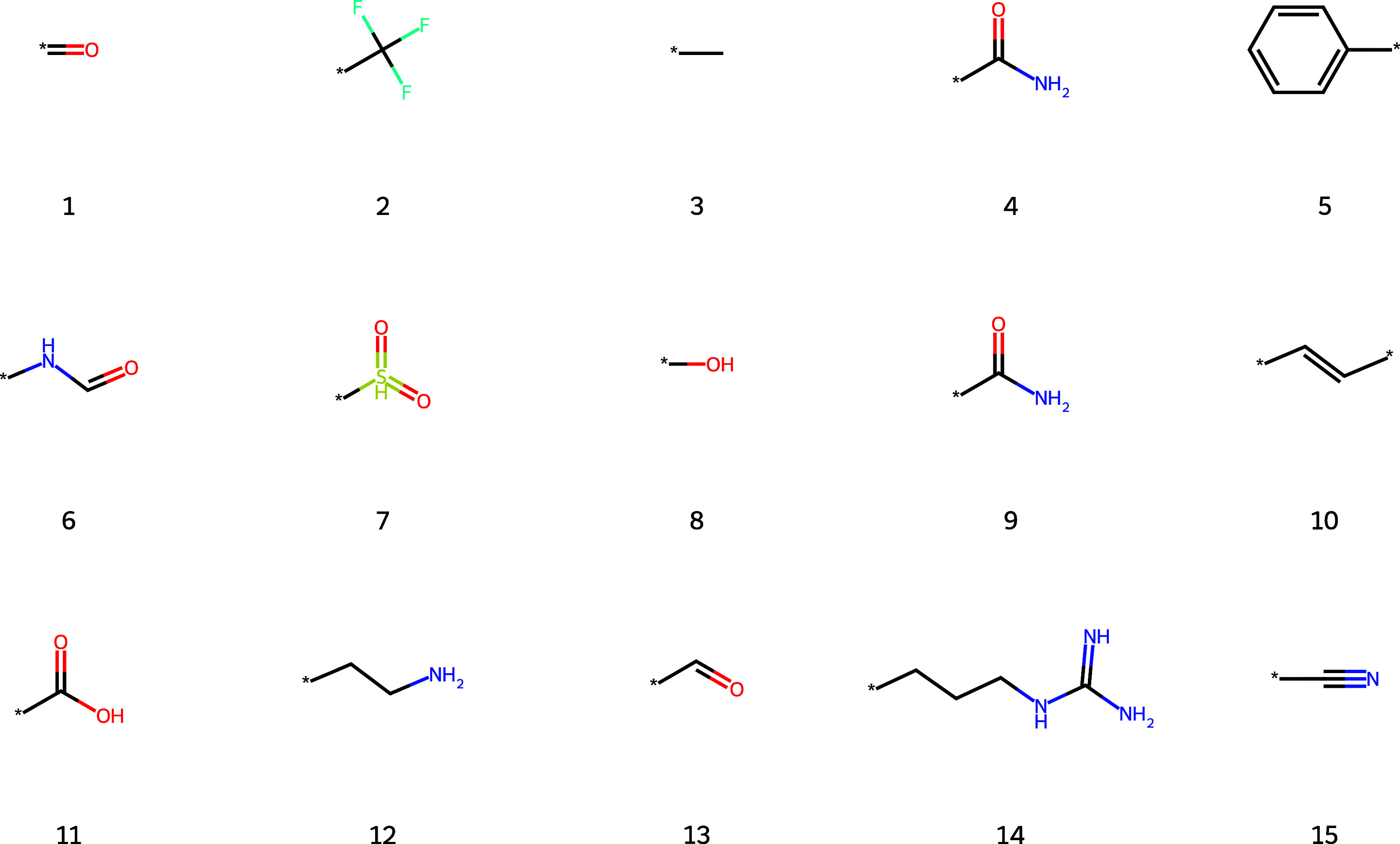
Top 15 substructures derived by the algorithm. Substructures are numbered in order of relative importance.

We now analyze subsets of the discovered groups: branches, small and large rings.

The top 40 branch substructures found by the algorithm are shown and numbered in Figure 8. Nearly all discovered branches are standard human functional groups. For example, the branches include carbonyl (16), methyl (17), alcohol (18) nitrile (19), fluoro (20), and amine (21) functional groups. As noted previously, due to the nature of the ChEMBL data set the number of peptide related functional groups is high. Specifically, there is an abundance of amino acid side chains and derivatives (substructures 22, 23, 26, 27, 28, 29, 30, 31, 32, 34, 38, 39). Other standard functional groups include benzyl (24, 44), trifluoromethyl (35), tertiary amines (37), morpholine (40), aromatic halides (46, 50), and the Boc protecting group (55). Interestingly, all halide substituents in the top 40 branches are in the para configuration. We were unaware a priori of the relative prevalence of ortho, meta, or para configurations.

**Figure 8:**
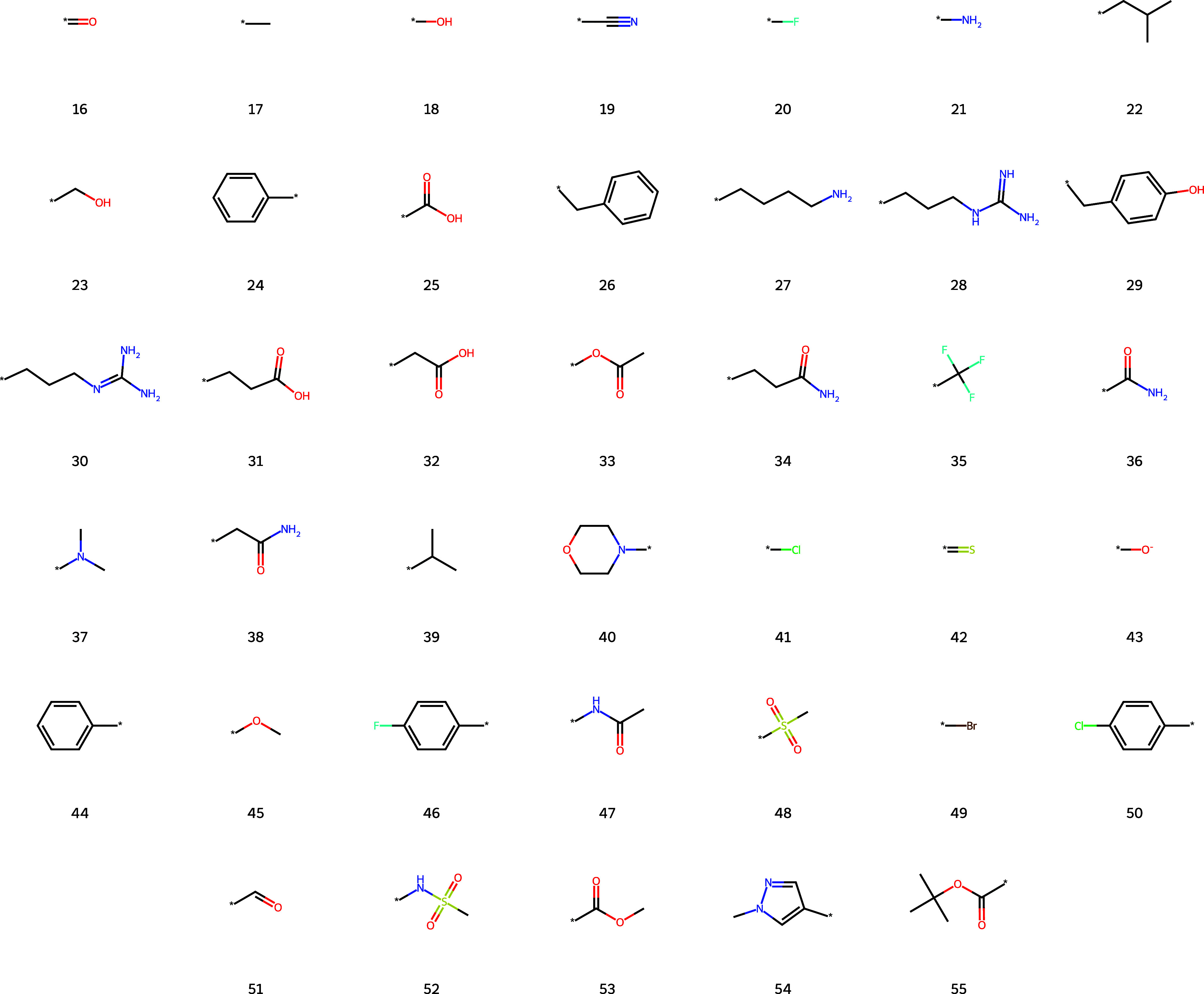
Top 40 substructures extracted as complete branches discovered by FGCompress. Substructures are numbered in order of relative importance.

The top 35 small ring containing substructures that may be expressed in fewer than 20 SMILES symbols are presented in Figure 9 below.

**Figure 9:**
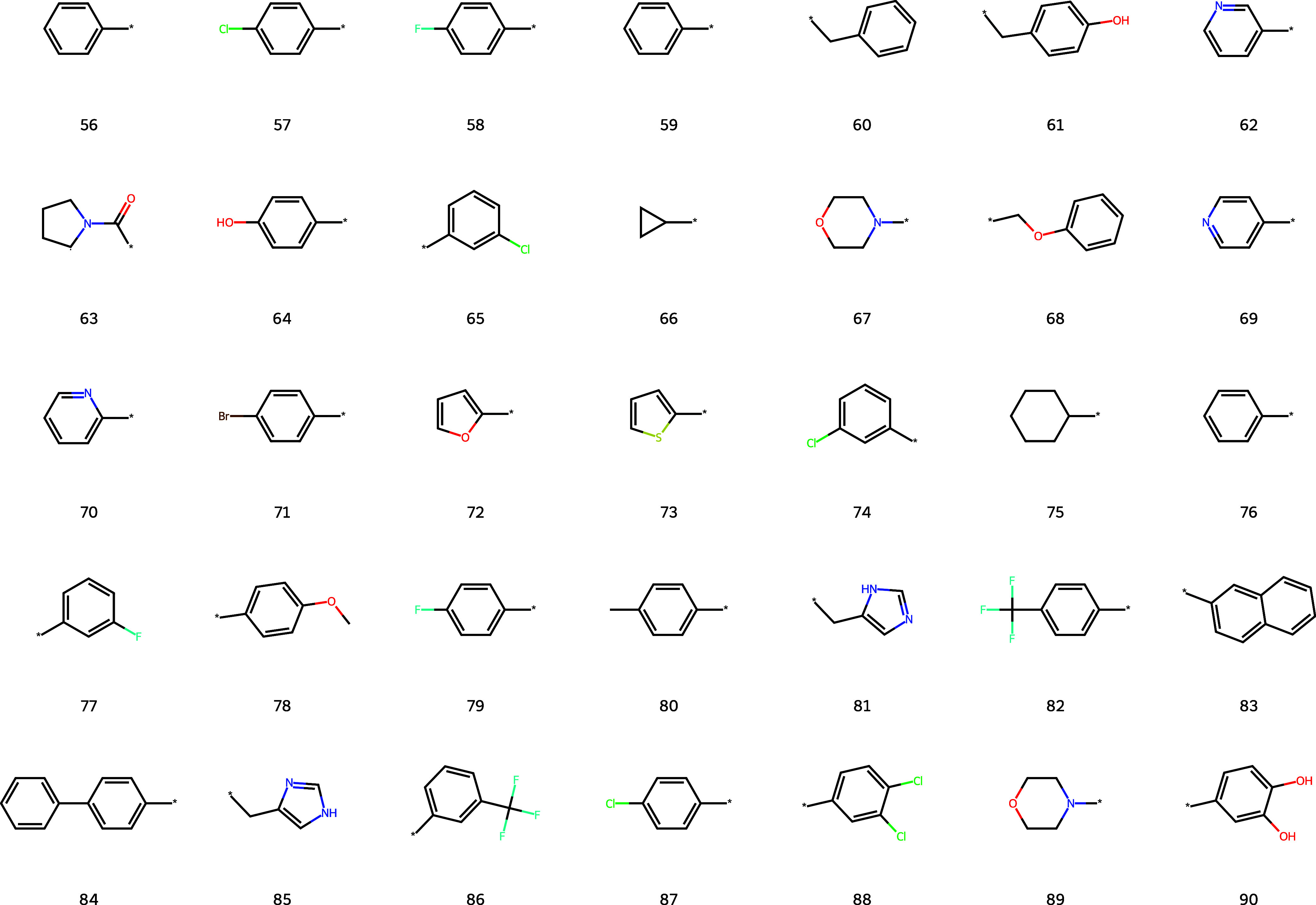
Substructures containing complete rings expressed in less than 20 symbols.

Most presented rings in Figure 9 are generally known substructures. As expected, the functional group benzene is ranked as the most important ring substructure by FGCompress. While many rings are known functional groups, such as morpholine (substructure 67), pyridine (substructure 70) furan (substructure 72), thiazole (substructure 73), napthalene (substructure 83), a large number may more fittingly be termed “substructures,” or combinations of primitive functional groups, such as substructures 68, 74, and 90.

Aside from the substituted benzene derivatives, the rings similarly contain several amino acid side chains (substructures 60 and 61). The stereochemistry of the disubstituted aromatic groups (substructures 88 and 90) appears reasonable, as the substitutions are positioned as far as possible from the connection point, thereby minimizing steric hindrance.

Larger rings that may be expressed in more than 19 symbols are shown in Figure 10. The substructures of Figure 10 are generally not themselves standard functional groups, but have a more specific biological function. We omit those substructures that are simple fragments of peptides. We also omit the discovered substring C­[C@@H]­1OP­(=O)­(O)­OC­[C@H]­1O­[C@@H], which, although technically valid, misrepresents the substructure in the original molecules. The original molecules contain two rings, both labeled with 1 to indicate each ring opening and closure. In the extracted substring, the two 1s are misinterpreted as belonging a single ring. We do not observe this specific issue in any other substring. Substructure *A* is a para-nitrobenzene which we do not find surprising. It is common knowledge that nitrobenzenes are ubiquitous in bioactive molecules. Substructure *B* forms part of a desosamine fragment, a central part of the pharmacophore of macrolide antibiotics.^32^ Substructure *C* is found in a number of compounds of the triterpene class such as lanosterol. Lanosterol is the precursor to cholesterol, the compound from which all animal and fungal steroids are derived and plays a role in maintaining lens health.^33,34^ A search on the ChEMBL database reveals that substructure *C* is present in 169 SMILES strings, and 85 distinct literature sources. These sources refer to investigations into compounds with promise in anticancer, neuroprotective, hepatoprotective, cataract therapy, and treatments of Chagas disease.^35–40^ Substructure *C* therefore features in a variety of biochemical explanations. Substructure *D* occurs in a number of antiviral therapies, including Zidovudine, Telbivudine, and Trifluridine.^41–43^ Substructure *E* features in the MCL-1 inhibitor AMG-176.^44^ Substructure *F* mainly occurs in a number of antifungal medications such as Fluconazole and Miconazole.^45,46^ In addition to those substructures shown in Figure 10, the amino acid sequence Leu-Arg-Glu-Phe-Tyr-Gly was also discovered. A search in the ChEMBL database reveals the sequence in peptides which bind Tissue Factor Pathway Inhibitor (TFPI).^47^

**Figure 10:**
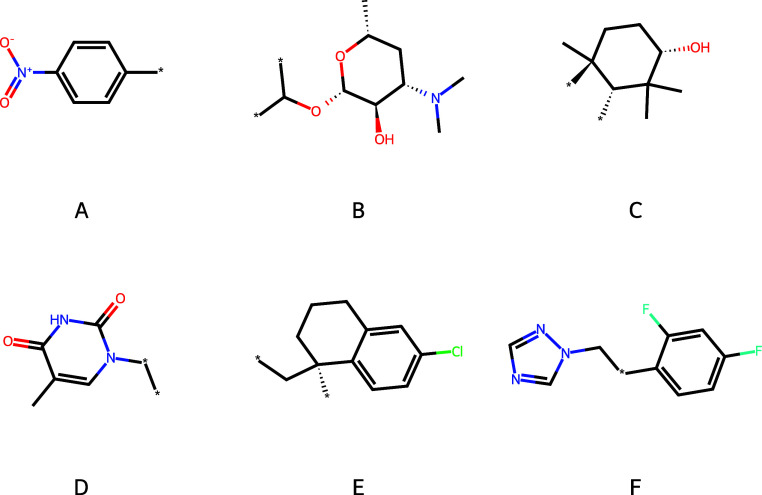
Large rings expressed in more than 19 symbols, amide containing ring fragments removed.

From our analysis we conclude that the answer to **Q1** is yes, that functional groups emerge from data compression of the ChEMBL data set. We additionally observe that FGCompress discovers data set specific functional groups, which correspond to more specific molecular behavior.

### Q2: Learning Benefit

Over the 24 data sets, we find that the Ridge Regression models trained using the MML87 fingerprint representation significantly outperform those trained using the MACCS fingerprint, Morgan fingerprint, and Neural embeddings (MolFormer-XL) representations at *p* < 0.05. The performance increase of the MML87 fingerprint was confirmed by Benjamini–Hochberg corrected significance tests at an overall *p* < 0.05. Specifically, we validate the claim that the MML87 fingerprint results in improved interpolative model predictions. The MML87 fingerprint outperforms both the MACCS and MolFormer-XL representations in 21 out of 24 cases and outperforms the equivalently sized Morgan fingerprint in 18 out of 24 cases. The results for each data set are summarized in Table 1.

**Table 1:** Mean Squared Error of the Ridge Regression Models Across Datasets and Fingerprint Representations. The Standard Error from the 5 Trials is Reported

data set	MML87	MACCS	molformer	morgan
B-raf	0.61 ± 0.00	0.72 ± 0.00	0.59 ± 0.01	**0.46** **±** **0.01**
ephrin	0.73 ± 0.01	0.78 ± 0.01	0.74 ± 0.02	**0.68** **±** **0.01**
caspase	**0.47** **±** **0.01**	0.65 ± 0.01	0.62 ± 0.01	0.53 ± 0.01
monoamine	0.75 ± 0.06	0.66 ± 0.01	0.69 ± 0.02	**0.59** **±** **0.01**
vanilloid	**0.60** **±** **0.01**	0.68 ± 0.01	0.72 ± 0.01	0.66 ± 0.02
acetylcholinesterase	**0.80** **±** **0.01**	1.06 ± 0.01	0.93 ± 0.01	0.83 ± 0.01
COX-1	**0.64** **±** **0.01**	0.70 ± 0.01	0.66 ± 0.01	0.82 ± 0.01
ABL1	**0.80** **±** **0.01**	0.92 ± 0.02	1.00 ± 0.02	0.89 ± 0.02
A2a	**0.68** **±** **0.02**	0.82 ± 0.02	0.74 ± 0.04	0.82 ± 0.03
COX-2	**0.80** **±** **0.01**	0.92 ± 0.01	0.80 ± 0.01	0.92 ± 0.02
dihydrofolate	**0.88** **±** **0.03**	1.01 ± 0.03	1.13 ± 0.04	1.04 ± 0.02
opioid	**0.73** **±** **0.01**	0.87 ± 0.01	0.83 ± 0.01	0.77 ± 0.02
glycogen	0.84 ± 0.04	0.96 ± 0.01	0.93 ± 0.01	**0.77** **±** **0.01**
erbB1	**0.70** **±** **0.00**	1.02 ± 0.01	0.76 ± 0.00	0.72 ± 0.00
LCK	**0.86** **±** **0.01**	1.06 ± 0.01	0.92 ± 0.01	0.93 ± 0.01
aurora-A	**0.82** **±** **0.01**	1.20 ± 0.01	0.92 ± 0.01	0.87 ± 0.01
glucocorticoid	**0.40** **±** **0.00**	0.52 ± 0.01	0.50 ± 0.01	0.48 ± 0.01
cannabinoid	**0.65** **±** **0.02**	0.94 ± 0.02	0.78 ± 0.01	0.81 ± 0.02
carbonic	0.57 ± 0.03	**0.51** **±** **0.01**	0.60 ± 0.01	0.70 ± 0.01
JAK2	0.66 ± 0.01	0.96 ± 0.01	0.72 ± 0.01	**0.62** **±** **0.01**
HERG	**0.43** **±** **0.01**	0.63 ± 0.01	0.50 ± 0.00	0.53 ± 0.00
coagulation	**0.85** **±** **0.01**	1.14 ± 0.02	1.13 ± 0.03	1.02 ± 0.01
estrogen	**0.49** **±** **0.00**	0.57 ± 0.01	0.67 ± 0.01	0.65 ± 0.00
dopamine	0.89 ± 0.03	**0.73** **±** **0.02**	0.84 ± 0.01	0.85 ± 0.02

These results suggest that the answer to **Q2** is yes, substructures that compress the data set are useful for learning and result in improved performance over the MACCS fingerprint on linear regression tasks. Note that we do not claim that the MML87 fingerprint is preferable for all learning models, just simple regression. For example, the random forest (RF) model is successful in QSAR tasks and insensitive to large numbers of low information features.^26,48^ Representations that enumerate vast numbers of substructures such as the Morgan fingerprint are instead likely to be beneficial for the RF model.

### Effect of Substring Length

We now describe the effect of the user-specified maximum substring length on the FGCompress algorithm. The substring length affects (i) the specific substructures extracted and (ii) the algorithm runtime. We describe each in turn.

### Effect on Extracted Substrings

There may be substring codebooks that are overall better for compression, but cannot not be found by smaller maximum substring lengths. As an example, consider compressing a data set consisting of only two molecules, represented as the SMILES strings {CCN­(=O), CN­(=O)­CC}. Consider two FGCompress runs with differing maximum substring lengths: run 1 has a maximum length of 2, while run 2 has a maximum length of 5. The two runs are shown in Figure 11 below.

**Figure 11:**
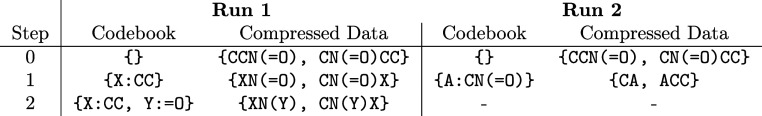
Effect of maximum substring length on discovered substructures.

Run 2 terminates at iteration 1: It finds the maximally compressing substring CN­(=O) and replaces it with the symbol *A* . Run 1 terminates at iteration 2: it finds the substrings CC and =O but fails to find the more compressing substring CN­(=O). The difference in codebooks occurs, because to add any expression in brackets to the codebook, such as (*Y*) (where *Y* denotes =O) one requires consideration of substructures of size greater than 2. There are two substrings of (*Y*) of length 2. The substrings are ((*Y* or *Y*)), neither of which are valid. Larger maximum substring lengths may be required to extract other meaningful patterns, such as those with nested brackets. Larger maximum substring lengths prevents the algorithm from missing large, compressing substrings.

### Effect on Runtime

The maximum substring length, however, also affects the runtime of the algorithm. The time complexity of enumerating all substrings up to length *N* increases exponentially with *N*. We plot the time taken per iteration for increasing maximum substring lengths 4, 8, 16, and 32 on the HERG and Acetylcholinesterase data sets in Figure 12 below. We plot the first 250 iterations in each case. The full codebook contains 3398 substructures. The smaller Acetylcholinesterase data set results in shorter runtimes, as there are fewer substrings to enumerate. We run the experiment on an M2MacBook Air (8 CPU cores, 16 GB RAM) using all CPU cores per experiment.

**Figure 12:**
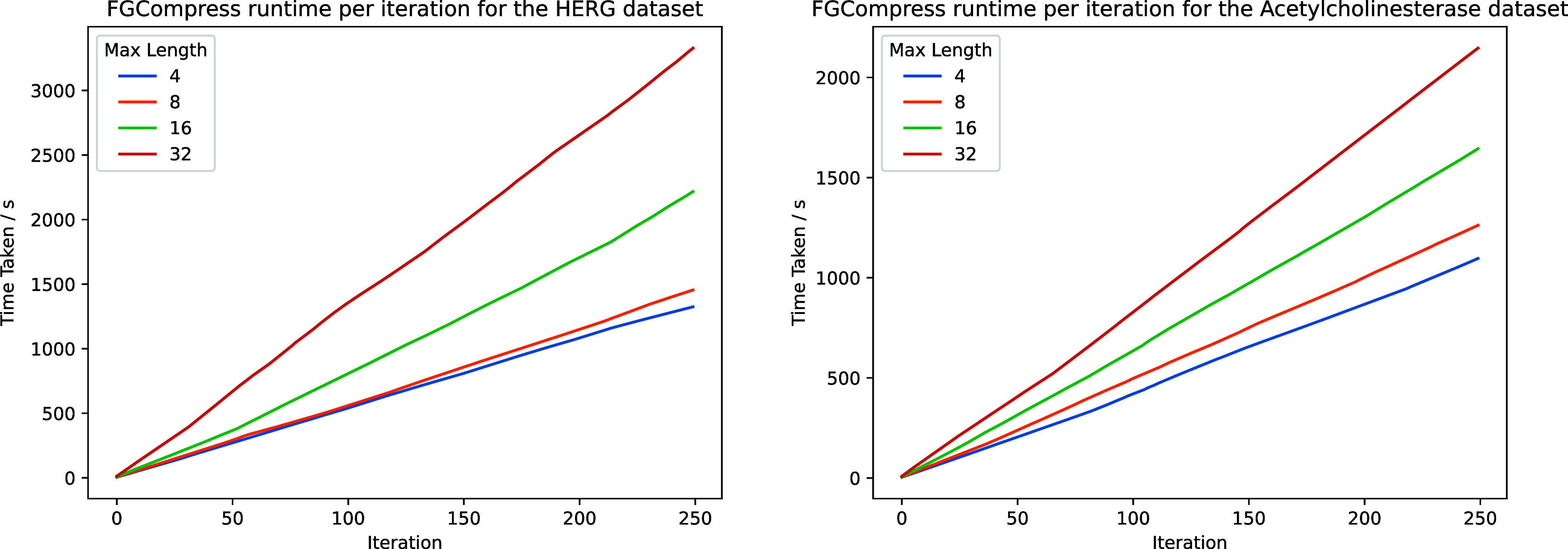
Runtime of the FGCompress over 250 iterations on the HERG (5207 molecules) and Acetylcholinesterase (3159 molecules) data sets. Larger substring lengths result in exponentially longer runtimes. Data sets with fewer molecules result in shorter runtimes.

## Conclusions

We showed that compressing chemistry results in the automatic identification of the standard set of human-identified functional groups, alongside several data set specific substructures. This work therefore provides computational validation that partitioning of molecules into functional groups is indeed a good general description of chemistry. We highlighted several interesting patterns that do not form part of the standard chemical functional group set and showed that several compressing functional groups correspond to specific biological function. Empirically, we have shown that fingerprints generated from the discovered substructures aid learning, and that ridge regression models trained on the MML87 fingerprints significantly outperform the MACCS and Morgan representations on 24 IC_50_ prediction tasks. The improved performance of the MML87 fingerprint over MACCS and Morgan is in spite of the reduced expressiveness of the MML87 fingerprint and comparable fewer substructures compared with the Morgan algorithm. Additionally, chemical fingerprints are often used for similarity scoring. Specifically, techniques like Taylor–Butina clustering leverage a chemical fingerprint to cluster molecules by similarity.^49^ Future work could also investigate the efficacy of the MML87 fingerprint in similarity scoring, comparing it to such techniques, and other common methods such as using Bemis–Murcko scaffolds.^50^

## Limitations

For computational tractability, we conducted our search procedure over SMILES strings. Consequently, our search space was restricted to only those substructures which may be represented as SMILES substrings. Unfortunately, SMILES substrings are a subset of the complete set of substructures within a molecule. Moreover, the same group in different compounds may be represented with different SMILES strings. We attempted to minimize this multiple representation issue by using canonical SMILES representations. Despite this issue, functional groups are still extracted by the algorithm because they occur at sufficiently high frequencies. Searching for subgraphs in graph molecule representations would be complete and remove representational redundancy, but computationally very expensive. Future work should explore a trade-off between the two such as lookahead searches, or hybrid representations. One could adapt recent advances in inductive logic programming (ILP), such as the use of constraint solvers to compress data sets to remedy this.^51–53^ We demonstrated that compressive substructures enhance predictive performance in learning tasks. However, certain features may be highly informative for explaining specific molecular properties even if they contribute little to overall data set compression. Moreover, widely used QSAR models such as random forests are relatively insensitive to the inclusion of many low-information features and often perform best with representations that enumerate large numbers of substructures, such as the Morgan fingerprint.^26^ The substructures identified by FGCompress therefore remain valuable and could serve as complementary features to enumerative fingerprints, particularly given that FGCompress imposes no restriction on maximum substructure size, unlike many conventional fingerprinting methods. The loss of connectivity information when constructing the fingerprint described in this report is a limitation of the FGFingerprinter method. We argue that this limitation is not necessarily problematic, as sophisticated machine learning methods, such as logic programming approaches, can easily incorporate connectivity information without much overhead. Additionally, the method could be used as an alternative tokenization scheme for neural network based modeling. When tokenizing a molecular data set, one simply may directly use the compressed data set representation as input, which preserves connectivity information. Further, the strong empirical results from the FGFingerprinter experiments lend evidence to the efficacy of the fingerprint, even when omitting the connectivity information.

## Supplementary Material



## Data Availability

All code to run the FGCompress algorithm is public and may be found at the github link: https://github.com/bars20/compressing-chemistry.

## Notes

DiscoveredGroups.txt: An exhaustive listing of the top 500 functional groups analyzed in this report. All groups are listed in the original SMILES string form (TXT)

## Abbreviations

MML: minimum message length
SMILES: simplified molecular input line entry system
MACCS: molecular ACCess system
